# Impact of U.S. Preventive Services Task Force lung cancer screening update on drivers of disparities in screening eligibility

**DOI:** 10.1002/cam4.5066

**Published:** 2022-07-24

**Authors:** Kristin G. Maki, Rajesh Talluri, Iakovos Toumazis, Sanjay Shete, Robert J. Volk

**Affiliations:** ^1^ Department of Health Services Research University of Texas MD Anderson Cancer Center Houston Texas USA; ^2^ Department of Data Science University of Mississippi Medical Center Jackson Mississippi USA; ^3^ Department of Biostatistics University of Texas MD Anderson Cancer Center Houston Texas USA; ^4^ Department of Epidemiology University of Texas MD Anderson Cancer Center Houston Texas USA; ^5^ Division of Cancer Prevention and Population Sciences University of Texas MD Anderson Cancer Center Houston Texas USA

**Keywords:** cancer screening eligibility, disparities, early detection, health services research, lung cancer screening

## Abstract

**Background:**

In 2021, the U.S. Preventive Services Task Force (USPSTF) updated its recommendation to expand lung cancer screening (LCS) eligibility and mitigate disparities. Although this increased the number of non‐White individuals who are eligible for LCS, the update's impact on drivers of disparities is less clear. This analysis focuses on racial disparities among Black individuals because members of this group disproportionately share late‐stage lung cancer diagnoses, despite typically having a lower intensity smoking history compared to non‐Hispanic White individuals.

**Methods:**

We used data from the National Health Interview Survey to examine the impact of the 2021 eligibility criteria on racial disparities by factors such as education, poverty, employment history, and insurance status. We also examined preventive care use and reasons for delaying medical care.

**Results:**

When comparing Black individuals and non‐Hispanic White individuals, our analyses show significant differences in who would be eligible for LCS: Those who do not have a high school diploma (28.7% vs. 17.0%, *p* = 0.002), are in poverty (26.2% vs. 14.9%, *p* < 0.001), and have not worked in the past 12 months (66.5% vs. 53.9%, *p* = 0.009). Further, our analyses also show that more Black individuals delayed medical care due to not having transportation (11.1% vs. 3.6%, *p* < 0.001) compared to non‐Hispanic White individuals.

**Conclusions:**

Our results suggest that despite increasing the number of Black individuals who are eligible for LCS, the 2021 USPSTF recommendation highlights ongoing socioeconomic disparities that need to be addressed to ensure equitable access.

## Graphical abstract

Our results suggest that the 2021 USPSTF recommendation on lung cancer screening may not reduce disparities associated with socioeconomic status. These factors include educational attainment, poverty level, employment history, and transportation for medical care.

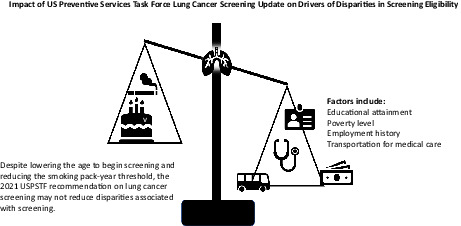

## 1 INTRODUCTION

In 2013, the U.S. Preventive Services Task Force (USPSTF) recommended annual lung cancer screening (LCS) for persons with a current or recent smoking history (i.e., quit within the past 15 years), aged 55–80 years, and have at least a 30 pack‐year smoking history.^1^ These criteria have been criticized as being too conservative for Black persons who smoke.^2^, ^3^ Despite less intense smoking histories, Black individuals who smoke are at a higher risk of lung cancer incidence^4^ and mortality than non‐Hispanic White individuals.^5^ Important factors for identifying Black persons who may benefit from LCS include having a current or history of smoking with a quit time greater than 15 years, fewer than 30 pack‐years, and ages less than 55 years.^3^ Among participants who were included in the National Lung Screening Trial, Black individuals were more likely to have fewer years of formal education, more comorbidities, and an increased likelihood of currently smoking compared to non‐Hispanic white individuals, highlighting other potential disparities.^5^

To address racial disparities, the USPSTF issued an updated LCS recommendation in 2021 that lowered the age to begin screening from 55 to 50 years and the minimum cumulative smoking exposure from 30 to 20 pack‐years.^6^ The effect of the 2021 USPSTF recommendation on the increase in Black individuals who are eligible for LCS has been supported by research using data from the Behavioral Risk Factor Surveillance System (BRFSS)^7^, ^8^ and the National Health Interview Survey (NHIS).^9^ However, despite the increase in eligibility among Black individuals, research with both BRFSS^7^, ^8^ and NHIS^9^ data has shown that the overall eligibility increase may still be the highest among non‐Hispanic White individuals. Indeed, the results of these analyses suggest that racial disparities will likely persist with the updated eligibility criteria.^8^

Moreover, despite the expanded screening eligibility, racial disparities in access to care will impact the effect of increased eligibility for LCS with the 2021 USPSTF recommendation. Specifically, racial differences in mortality are more equal if the stage of diagnosis and access to care is considered.^10^ Although this finding is not new, few advances have been made in mitigating the disparity.^11^ Prior work has also shown that Black individuals more frequently cite concern about screening convenience and cost of LCS than non‐Hispanic white individuals.^12^

Racial disparities extend beyond screening initiation. For instance, there are differences in adherence to annual LCS, with lower adherence observed in certain groups including individuals who currently smoke, have less than a college education, individuals who are racial minorities, and those who are less than 65 years old.^13^ Similarly, one study found that Black individuals who had attained more than a high school education had a higher likelihood of adhering to annual LCS than non‐Hispanic White individuals who had less than a high school education.^12^

We expand on this discussion by examining the 2021 USPSTF update's effect on socioeconomic disparities related to access to care. Specifically, due to the racial inequities, we examined disparities between Black individuals and non‐Hispanic White individuals. We focused our analysis on drivers of disparities by assessing eligibility among individuals with less than a high school diploma, without health insurance, and who are below the poverty line. We also examined variables that reflect access to care, including health insurance coverage, having a regular place to receive preventive care, and factors that caused a delay in medical care.

These are crucial aspects to consider in conjunction with LCS eligibility because factors such as lower socioeconomic status and lack of trust in the healthcare provider are associated with later stage diagnosis of lung cancer.^14^ Further, underserved individuals have an increased likelihood of not having a primary care physician.^14^ These factors are compounded with systemic racial inequities. For instance, one study's results showed that Black individuals were diagnosed at a later stage of lung cancer than other racial groups regardless of insurance type (excluding Medicaid).^15^

Our objectives are: (i) expand the examination of the update's impact on racial disparities in LCS; and (ii) address a fundamental concern about access to care, operationalized as having health insurance, using preventive care, and reasons for delayed medical care.

## 2 METHODS

We analyzed the 2015 National Health Interview Survey data,^16^ which are the most recent data including the cancer control supplement. This survey is administered to the noninstitutionalized population of the U.S. by the National Center for Health Statistics. The Research Ethics Review Board of the National Center for Health Statistics approved the NHIS cycle used in our secondary analysis and NHIS respondents provided consent prior to participation in this survey. Additional details regarding the NHIS survey and sampling methods can be found at https://www.cdc.gov/nchs/nhis/about_nhis.htm. Because the NHIS data are publicly available and anonymized, our secondary data analysis was exempt from institutional review board approval, in accordance with 45 CFR §46.^17^ The data that support the findings of this study are openly available from the National Center for Health Statistics at https://www.cdc.gov/nchs/nhis/nhis_2015_data_release.htm.

In addition to including variables for key factors such as poverty level, educational attainment, and health insurance status, this data source provides more complete information about persons who no longer smoke (i.e., the ability to calculate pack‐years) than other years' data without the supplement. It is representative of adults in the United States. Data from the 2015 NHIS, as well as other sources, informed the USPSTF update.^6^

We included participants who met the eligibility criteria of the 2013 and 2021 USPSTF recommendations; the adult U.S. population of adults 50 to 80 years old is the reference group. We examined: race/ethnicity, geographic region, education level, poverty, employment history, insurance status, use of preventive care, and reasons for delaying care. We examined proportions within the eligible population (i.e., 50–80 years old, ≥20 pack‐year history). The data were weighted to reflect the civilian, noninstitutionalized U.S. population at the time of the survey.^18^

We conducted a subgroup analysis to estimate drivers of disparities (i.e., education, poverty, employment, health insurance, preventive care, and reasons for delaying medical care) by comparing differences in proportions of non‐Hispanic White and non‐Hispanic Black participants who may be eligible for LCS with the 2021 USPSTF recommendation. We tested significance with Pearson's chi‐square using the survey^19^ package in R. All statistical procedures accounted for the complex sample design and were assessed with a two‐tailed test. Cases with missing data were omitted from analyses.

## 3 RESULTS

Participants for this analysis included adults who would meet age and smoking history criteria for USPSTF 2013 (*n* = 1325; weighted *n* = 7,855,300), and 2021 update (*n* = 2318; weighted *n* = 14,247,383) recommendations. Overall, our results show increases in eligibility (Table 1), aligning with other analyses.^7^, ^9^

**TABLE 1:** Estimated number of individuals eligible for lung cancer screening with USPSTF 2013 and 2021 recommendations

	USPSTF 2013		USPSTF 2021		% Relative increase
	*n* (%)	95% CI	*n* (%)	95% CI	
Estimated number eligible for LCS	7,855,300 (100%)	7,277,312–8,433,288	14,247,383 (100%)	13,461,620–15,033,146	81.4
Sex					
Male	4,516,657 (57.5%)	4,063,799–4,969,515	7,895,284 (55.4%)	7,292,622–8,497,946	74.8
Female	3,338,643 (42.5%)	2,963,848–3,713,438	6,352,099 (44.6%)	5,836,622–6,867,576	90.3
Race and ethnicity					
Hispanic	277,418 (3.5%)	182,658–372,178	591,030 (4.1%)	452,522–729,538	113.0
Non‐Hispanic White	6,833,710 (87.0%)	6,298,882–7,368,538	12,158,729 (83.7%)	11,415,700–12,901,758	77.9
Non‐Hispanic Black	538,271 (6.9%)	428,394–648,148	1,116,653 (7.7%)	951,111–1,282,195	107.5
Non‐Hispanic Asian	157,408 (2.0%)	67,400–247,416	538,271 (3.7%)	153,027–366,523	242.0
All other race and ethnic groups	48,493 (0.6%)	13,821–83,165	121,196 (0.8%)	56,803–185,589	149.9
Educational attainment					
Less than high school diploma/GED	1,572,707 (20.0%)	1,283,351–1,862,063	2,711,036 (19.1%)	2,331,751–3,090,321	72.4
High school diploma and above	6,272,003 (80.0%)	5,743,668–6,800,338	11,509,652 (80.9%)	10,809,045–12,210,259	83.5
Region					
Northeast	1,188,421 (18.1%)	925,393–1,451,450	2,457,151 (17.2%)	2,046,295–2,868,007	106.8
Midwest	2,080,986 (29.7%)	1,783,397–2,378,575	3,740,965 (26.3%)	3,383,181–4,098,749	79.8
South	3,207,448 (44.4%)	2,849,760–3,565,136	5,664,008 (39.8%)	5,165,340–6,162,676	76.6
West	1,378,445 (20.1%)	1,164,599–1,592,291	2,385,259 (16.7%)	2,118,957–2,651,561	73.0
Income to poverty ratio					
≥1 (i.e., above poverty level)	4,591,780 (87.6%)	4,136,471–5,047,089	7,943,944 (84.0%)	7,332,187–8,555,701	73.0
<1 (i.e., poverty level or below)	822,549 (18.0%)	665,434–979,664	1,513,357 (16.0%)	1,281,157–1,745,557	84.0
Employment history					
Work currently or in past 12 months	3,084,412 (39.3%)	2,704,228–2,704,228	6,424,077 (45.1%)	5,877,936–6,970,218	108.3
Have not worked in past 12 months	4,768,591 (60.7%)	4,333,778–5,203,404	7,814,826 (54.9%)	7,221,070–8,408,582	63.9
Health insurance status					
Insured	7,346,100 (93.6%)	6,778,725–7,913,476	13,177,495 (92.6%)	12,412,458–13,942,532	79.4
Uninsured	499,857 (6.4%)	359,017–640,697	1,052,394 (7.4%)	842,148–1,262,640	110.5
Health insurance subcategories (individuals <65 years)					
Private	2,572,038 (57.6%)	2,216,304–2,927,773	5,395,081 (57.1%)	4,876,217–5,913,945	109.8
Medicaid and other public	693,051 (15.5%)	513,212–872,890	1,715,428 (18.2%)	1,405,381–2,025,475	147.5
Other coverage	711,954 (15.9%)	536,100–887,809	1,297,275 (13.7%)	1,035,368–1,559,182	82.2
No insurance	488,384 (10.9%)	348,423–628,345	1,038,976 (11.0%)	829,487–1,248,465	112.7
Health insurance subcategories (individuals 65+ years)					
Private	1,276,843 (32.9%)	1,032,761–1,520,925	1,864,919 (33.7%)	1,559,561–2,170,277	46.1
Dual eligible	301,475 (8.9%)	177,983–424,967	417,182 (8.7%)	276,166–558,198	38.4
Medicare only	1,410,068 (24.1%)	1,185,566–1,634,570	1,956,333 (23.4%)	1,704,083–2,208,583	38.7
Other coverage	375,496 (22.6%)	261,788–489,204	526,102 (22.9%)	397,173–655,031	40.1
Uninsured	11,473 (11.1%)	−4235–27,181	13,418 (11.0%)	−2592–29,428	17.0
Use of preventive care					
Does not get preventive care	539,298 (49.5%)	367,722–710,874	1,025,215 (49.3%)	813,331–1,237,099	90.1
Clinic or health center	134,094 (12.3%)	59,889–208,299	229,752 (11.0%)	132,939–326,565	71.3
Doctor's office or HMO	243,494 (22.4%)	121,906–365,082	530,547 (25.5%)	361,377–699,717	117.9
Hospital emergency room	6927 (0.6%)	−3188–17,042	39,132 (1.9%)	405–77,859	464.9
Hospital outpatient	13,856 (1.3%)	−4380–32,092	28,088 (1.4%)	−1871–58,047	102.7
Some other place	28,333 (2.6%)	−3935–60,601	59,929 (2.9%)	12,665–107,193	111.5
Does not go to one place most often	123,446 (11.3%)	50,814–196,078	167,288 (8.0%)	86,412–248,164	35.5
Reasons for delaying medical care in the past 12 months					
Could not get through on phone (Yes)	221,232 (2.8%)	123,238–319,226	430,459 (3.0%)	284,849–576,069	94.6
Could not get through on phone (No)	7,616,591 (97.2%)	7,041,330–8,191,852	13,780,929 (97.0%)	12,993,818–14,568,040	80.9
Could not get appointment soon enough (Yes)	518,591 (6.6%)	378,942–658,241	1,001,343 (7.0%)	791,033–1,211,653	93.1
Could not get appointment soon enough (No)	7,319,232 (93.4%)	6,772,201–7,866,263	13,206,395 (93.0%)	12,449,759–13,963,031	80.4
Wait too long in doctor's office (Yes)	374,537 (4.8%)	252,663–496,411	763,318 (5.4%)	594,333–932,303	103.8
Wait too long in doctor's office (No)	7,463,286 (95.2%)	6,895,205–8,031,367	13,448,070 (94.6%)	12,676,526–14,219,614	80.2
Not open when you could go (Yes)	108,990 (1.4%)	48,964–169,016	390,387 (2.7%)	249,867–530,907	258.2
Not open when you could go (No)	7,728,833 (98.6%)	7,156,118–8,301,548	13,820,287 (97.3%)	13,050,750–14,589,824	78.8
No transportation (Yes)	286,416 (3.7%)	151,571–421,261	637,027 (4.5%)	449,169–824,886	122.4
No transportation (No)	7,551,407 (96.3%)	6,978,651–8,124,163	13,573,647 (95.5%)	12,797,613–14,349,681	79.7

Abbreviations: CI, confidence interval; LCS, lung cancer screening; USPSTF, U.S. Preventive Services Task Force.

In our subgroup analysis (Table 2), we found significant differences in several important drivers of disparities among the respondents who would be eligible for LCS with the updated recommendation. Specifically, there was a higher proportion of Black respondents who did not have a high school diploma (*p* = 0.002), whose income to poverty ratio was below 1 (*p* < 0.001), and who had not worked in the past 12 months (*p* = 0.009) compared to White respondents. Regarding insurance status, there was a significant difference in the type of health insurance that non‐Hispanic White and non‐Hispanic Black respondents had for both individuals under 65 years (*p* = 0.045) and those 65 years and older (*p* = 0.088). Similarly, there was a difference in where non‐Hispanic White and non‐Hispanic Black respondents obtained preventive care (*p* = 0.066). Finally, the proportion of Black respondents who had delayed medical care due to a lack of transportation was higher than White respondents (*p* < 0.001). To provide additional context about the impact of the updated recommendation on racial disparities, a summary of our subgroups' estimated increases is provided in Table S1.

**TABLE 2:** Comparison of racial disparities among respondents eligible for LCS with 2021 USPSTF recommendation

	Non‐Hispanic White				Non‐Hispanic Black				*p*‐value^a^
	%	*SE*	95% CI		%	*SE*	95% CI		
Male	54.5	1.6	51.46	57.55	59.8	3.7	52.56	67.04	0.190
Female	45.5	1.6	42.45	48.54	40.2	3.7	32.96	47.44	
Less than high school/GED attained	17.0	1.3	14.6	19.5	28.7	4.1	20.7	36.8	0.002
Income to poverty ratio <1	14.9	1.3	12.3	17.5	26.2	3.7	18.9	33.5	<0.001
Have not worked in past 12 months	53.9	1.6	50.7	57.0	66.5	4.3	58.0	75.0	0.009
Health insurance (individuals <65 years)									0.045
Private	59.4	2.0	55.5	63.3	44.5	5.6	33.5	55.4	
Medicaid and other public	16.6	1.7	13.3	19.8	26.0	5.1	16.0	36.1	
Other coverage	13.8	1.4	11.0	16.6	16.2	4.0	8.3	24.1	
No insurance	10.2	1.1	8.0	12.4	13.3	3.1	7.2	19.4	
Health insurance (individuals 65+ years)									0.088
Private	35.4	2.5	30.6	40.2	18.9	4.5	10.1	27.8	
Dual eligible	7.4	1.6	4.3	10.5	14.0	4.2	5.7	22.2	
Medicare only	24.1	2.1	19.9	28.2	24.6	5.4	14.0	35.2	
Other coverage	22.1	1.9	18.4	25.8	29.9	5.7	18.7	41.1	
Uninsured	10.9	1.6	7.7	14.0	12.5	4.3	4.1	21.0	
Preventive care									0.066
Does not get preventive care	49.4	4.6	40.4	58.5	41.1	10.9	19.7	62.5	
Clinic or health center	10.8	2.6	5.8	15.9	12.8	7.1	−1.1	26.7	
Doctor's office or HMO	26.1	3.9	18.4	33.8	23.0	9.8	3.8	42.1	
Hospital emergency room	1.7	1.1	−0.5	3.8	6.0	3.6	−1.1	13.1	
Hospital outpatient	0.4	0.4	−0.3	1.1	7.0	6.9	−6.4	20.5	
Some other place	3.0	1.3	0.4	5.6	4.9	4.8	−4.5	14.4	
Does not go to one place most often	8.5	2.2	4.2	12.8	5.1	3.1	−1.0	11.2	
Reasons for delaying medical care in the past 12 months									
Could not get through on phone (Y)	2.9	0.6	1.8	4.0	5.7	2.5	0.8	10.6	0.162
Could not get appointment soon enough (Y)	6.6	0.8	5.1	8.1	10.5	3.2	4.3	16.7	0.154
Wait too long in doctor's office (Y)	4.9	0.7	3.6	6.1	10.4	3.0	4.6	16.2	0.023
Not open when you could go (Y)	2.9	0.6	1.8	4.0	3.4	1.7	0.0	6.8	0.766
No transportation (Y)	3.6	0.7	2.3	4.9	11.1	3.1	5.0	17.2	<0.001

*Note*: Analysis included individuals who would be eligible for LCS using the 2021 USPSTF recommendation.

Abbreviations: CI, confidence interval; LCS, lung cancer screening; SE, standard error; USPSTF, U.S. Preventive Services Task Force.

^a^ *p*‐value for Rao‐Scott adjusted Pearson chi‐square.

## 4 DISCUSSION

The 2021 USPSTF recommendation update will dramatically increase the number of adults in the U.S. who are eligible for LCS, yet this may not address drivers of inequities related to LCS. Regarding sheer increases in eligibility, the impact will likely be the greatest among Black individuals. With optimal uptake and adherence, lowering LCS criteria to 20 pack‐years could help mitigate racial disparities in lung cancer mortality.^2^ However, additional work will be needed because LCS adherence is markedly lower in community settings than what was seen in clinical trials.^13^ Despite the increases in the proportion of individuals who are now eligible for LCS based on the updated recommendation, further work is needed to mitigate inequities in access.^7^, ^20^, ^21^

Our work contributes to the analysis of the 2021 update's potential impact by examining drivers of disparities in relation to the lower age and pack‐year criteria by elucidating drivers of inequities in care. Specifically, our findings show potential gaps, particularly among non‐Hispanic Black individuals who have lower levels of education, income, and employment. These factors are important to consider within the context of exacerbating disparities that may compound upon one another. Specifically, rates of tobacco use are higher among individuals whose education level is less than high school, compared to individuals who have completed additional education.^22^ Further, factors such as health literacy, lack of knowledge about lung cancer, cultural factors, and mistrust due to systemic barriers and mistreatment have all contributed to racial disparities in LCS and mortality among Black individuals.^10^ In addition, individuals with lower levels of education, who are uninsured or underinsured, who have lower levels of health literacy, and who belong to a racialized group are less likely to navigate the healthcare system.^10^ This is further compounded with the relatively inaccessible content of LCS programs' materials^23^ as well as educational materials that are available online.^24^ For instance, an analysis of 257 LCS program websites found several areas of concern, including reading levels consistently being rated above the average reading level of adults in the US, as well as variable information on eligibility criteria, potential costs, and the logistics related to screening.^23^ Updating these materials to meet recommendations may help in boosting accessibility for patients.^23^ Patient navigators who are culturally competent may be another way to help mitigate this challenge.^10^, ^25^

In addition to these socioeconomic factors, our analysis suggests that access to care may further exacerbate these individuals' disparities regarding LCS. For instance, our examination of the health insurance status of non‐Hispanic White individuals and non‐Hispanic Black individuals suggests that a lower percentage of Black individuals have private insurance compared to White individuals (44.5% vs. 59.4%), whereas a higher percentage is insured through Medicaid (26.0% vs. 16.6%). This discrepancy elucidates potential disparities in access to care due to LCS coverage being determined at the state level for Medicaid beneficiaries.^11^, ^26^ Additional barriers may be in place beyond Medicaid coverage.^27^

Limited transportation may also be an important consideration in relation to access to care. Our results showed that more non‐Hispanic Black individuals who would be eligible for LCS with the 2021 USPSTF recommendation had delayed medical care within the past 12 months compared to non‐Hispanic White individuals. This is an important finding due to the already documented disparity in geographic distribution of screening facilities. Specifically, prior work has shown clusters of screening facilities in areas that have greater resources and population density. For instance, Niranjan et al.^28^ estimated the geographic distance for individuals to get to LCS Center of Excellence (SCOE) locations. Within this analysis, geographic proximity was operationalized as a 60‐minute drive; the results showed a cluster of SCOEs in the Northeastern and Midwestern regions, with a broad band of high‐mortality counties spread throughout the Appalachian and Southeastern US with limited access to SCOEs.^28^

Similar work has suggested that approximately 5% of the screening eligible population is located more than 40 miles from a screening facility^29^ and accessibility to screening locations is associated with population density.^30^ Moreover, an analysis of census tract‐level socioeconomic characteristics and distance to ACR‐accredited computed tomography (CT) facilities showed increased driving distances to CT facilities for census tracts with higher proportions of individuals who are uninsured, have Medicaid, and have less than a high school degree.^31^ Taken together, these studies' results underscore the importance of access to transportation for use of LCS. Potential mitigation strategies outside of expanding the number of screening facilities may include the use of mobile screening units, which has been shown as a promising approach for reaching underserved populations without compromising screening quality.^32^, ^33^, ^34^ Alternatively, expanding centralized telehealth models has also been suggested as an additional strategy for addressing geographic hurdles.^26^, ^35^

Limitations of this analysis include estimating eligibility from NHIS data; variations in sampling and nonresponse are minimized with weighting.^16^ Due to the availability of pertinent variables, we used data from 2015. Finally, we did not include clinical considerations that could impact eligibility because those would be determined in consultation with a healthcare provider.

The USPSTF 2021 update will increase the proportion of adults in the U.S. eligible for LCS, particularly Black individuals. This may allow more individuals who share a disproportionate burden of later stage diagnosis, despite lower tobacco use, to undergo LCS earlier. However, as other work has also suggested, this adjustment will likely not mitigate the disparities that exist in LCS and mortality.^7^, ^8^ Additional adjustments are needed in order to address socioeconomic disparities in LCS eligibility and care. For instance, at the professional recommendation level, individual risk assessments may be incorporated into screening eligibility and decision‐making to help address a broader risk profile^26^, ^36^ that may include life expectancy and past screening results.^37^ At the provider level, further awareness of LCS and the eligibility criteria may be helpful in identifying patients for whom screening may be an option, along with gaining competence in shared decision‐making and tobacco cessation counseling. Facilitating these discussions in a manner that does not stigmatize patients may also be helpful in addressing disparities related to lung cancer.^26^ Additionally, increasing accessibility through mobile clinics, telehealth, and other innovations in care delivery may help to reduce some barriers to screening. At the patient level, integrating patient navigators, interventions to address health literacy, and efforts to reduce medical mistrust may help in reducing barriers to screening and improving lung cancer outcomes among minority populations.^10^, ^11^

## AUTHOR CONTRIBUTIONS

*Concept and design*: All authors. *Acquisition, analysis, interpretation of data*: Rajesh Talluri and Kristin G. Maki. *Drafting of manuscript*: Kristin G. Maki. *Critical revision of manuscript*: All authors. *Statistical analysis*: Rajesh Talluri and Kristin G. Maki. *Supervision*: Robert J. Volk and Sanjay Shete.

## FUNDING INFORMATION

This study was supported by grants from the Cancer Prevention and Research Institute of Texas (CPRIT; RP190210), and The University of Texas MD Anderson's Cancer Center Support Grant funded from NIH/NCI under award number P30CA016672 (using the Shared Decision Making Core and Clinical Protocol and Data Management System). It was also supported in part by a cancer prevention fellowship for Kristin Maki, supported by the Cancer Prevention and Research Institute of Texas grant award, RP170259, Shine Chang, PhD, Principal Investigator and by the MD Anderson Cancer Center Support Grant, CA016672, funded by the National Cancer Institute.

## CONFLICTS OF INTEREST

The authors do not have conflicts of interest to report.

## Supporting information


Table S1

## Data Availability

The data that support the findings of this study are openly available from the National Center for Health Statistics at https://www.cdc.gov/nchs/nhis/nhis_2015_data_release.htm.
