# Supplementary material for: Impact of U.S. Preventive Services Task Force lung cancer screening update on drivers of disparities in screening eligibility
Source: Cancer Med. 2022 Jul 24;12(4):4647–54. doi: 10.1002/cam4.5066 (PMC9972155; doi:10.1002/cam4.5066)
Supplement: Supplementary file 1 — Table S1 [file CAM4-12-4647-s001.docx]

| ***Supplemental table 1***. Comparison of Non-Hispanic White and Non-Hispanic Black Individuals’ LCS Eligibility with USPSTF 2013 and 2021 Recommendations | | | | | | | | |
| --- | --- | --- | --- | --- | --- | --- | --- | --- |
|  | **USPSTF 2013** | | | | **USPSTF 2021** | | | |
|  | **Non-Hispanic White** | | **Non-Hispanic Black** | | **Non-Hispanic White** | | **Non-Hispanic Black** | |
|  | **n (%)** | **95% CI** | **n (%)** | **95% CI** | **n (%)** | **95% CI** | **n (%)** | **95% CI** |
| **Total Eligible** | 6,833,710 | 6,298,882 – 7,368,538 | 538,271 | 428,394 – 648,148 | 12,158,729 | 11,415,700 – 12,901,758 | 1,116,653 | 951,112 – 1,282,195 |
| Sex | | | | | | | | |
| Male | 3,870,212 (56.6%) | 3,447,215 – 4,293,209 | 316,566 (58.8%) | 228,403 – 404,729 | 6,627,583 (54.5%) | 6,063,092 – 7,192,074 | 667,749 (59.8%) | 535,694 – 799,804 |
| Female | 2,963,498 (43.4%) | 2,616,675 – 3,310,321 | 221,705 (41.2%) | 151,386 – 292,024 | 5,531,146 (45.5%) | 5,044,643 – 6,017,649 | 448,904 (40.2%) | 347,796 – 550,012 |
| Educational Attainment | | | | | | | | |
| < High school | 1,245,391 (18.2%) | 983,138 – 1,507,644 | 163,448 (30.4%) | 99,115 – 227,781 | 2,070,076 (17.0%) | 1,740,954 – 2,399,198 | 316,387 (28.7%) | 216,786 – 415,988 |
| ≥ High school | 5,588,319 (81.8%) | 5,090,777 – 6,085,861 | 374,823 (69.6%) | 278,737 – 470,909 | 10,087,176 (83.0%) | 9,408,407 – 10,765,945 | 785,638 (71.3%) | 637,368 – 933,908 |
| Geographic Region | | | | | | | | |
| Northeast | 1,047,104 (15.3%) | 800,223 – 1,293,985 | 73,191 (13.6%) | 24,576 – 121,806 | 2,171,088 (17.9%) | 1,770,326 – 2,571,850 | 141,758 (12.7%) | 83,528 – 199,988 |
| Midwest | 1,923,438 (28.1%) | 1,650,743 – 2,196,133 | 106,175 (19.7%) | 64,214 – 148,136 | 3,413,550 (28.1%) | 3,079,116 – 3,747,984 | 208,770 (18.7%) | 129,584 – 287,956 |
| South | 2,739,208 (40.1%) | 2,410,094 – 3,068,322 | 294,719 (54.8%) | 216,219 – 373,219 | 4,642,629 (38.2%) | 4,175,611 – 5,109,647 | 654,507 (58.6%) | 542,324 – 766,690 |
| West | 1,123,960 (16.4%) | 925,021 – 1,322,899 | 64,186 (11.9%) | 21,918 – 106,454 | 1,931,462 (15.9%) | 1,685,730 – 2,177,194 | 111,618 (10.0%) | 39,799 – 183,437 |
| Income to Poverty Ratio (higher value indicates higher income) | | | | | | | | |
| ≥ 1 | 4,050,539 (85.6%) | 3,627,565 – 4,473,514 | 302,092 (74.5%) | 221,231 – 382,953 | 6,918,395 (85.1%) | 6,338,095 – 7,498,695 | 564,634 (73.8%) | 449,507 – 679,761 |
| < 1 | 682,968 (14.4%) | 535,727 – 830,209 | 103,257 (25.5%) | 59,208 – 147,306 | 1,214,012 (14.9%) | 996,920 – 1,431,104 | 200,503 (26.2%) | 136,139 – 264,867 |
| Employment Status in the Past 12 Months | | | | | | | | |
| Employed | 2,675,609 (39.2%) | 2,323,639 – 3,027,579 | 179,317 (33.3%) | 110,378 – 248,256 | 5,604,596 (46.1%) | 5,089,486 – 6,119,706 | 373,234 (33.5%) | 261,954 – 484,514 |
| Unemployed | 4,155,804 (60.8%) | 3,745,928 – 4,565,680 | 358,954 (66.7%) | 262,824 – 455,084 | 6,548,178 (53.9%) | 5,994,330 – 7,102,026 | 740,894 (66.5%) | 599,893 – 881,896 |
| Health Insurance Status | | | | | | | | |
| Insured | 6,418,999 (93.9%) | 5,886,636 – 6,951,362 | 510,471 (94.8%) | 404,106 – 616,836 | 11,315,726 (93.2%) | 10,594,549 – 12,036,903 | 1,020,585 (91.7%) | 862,064 – 11,79,106 |
| Uninsured | 414,711 (6.1%) | 285,330 – 544,092 | 27,800 (5.2%) | 7,135 – 48,465 | 829,628 (6.8%) | 645,893 – 1,013,363 | 91,949 (8.3%) | 46,759 – 137,139 |
| Health Insurance Subcategories (individuals < 65 years of age) | | | | | | | | |
| Private | 2,277,990 (58.7%) | 1,945,083 – 2,610,897 | 156,421 (52.7%) | 80,865 – 231,977 | 4,793,273 (59.4%) | 4,306,762 – 5,279,784 | 307,623 (44.5%) | 197,485 – 417,761 |
| Public | 547,866 (14.1%) | 390,616 – 705,116 | 64,600 (21.7%) | 28,094 – 101,106 | 1,336,339 (16.6%) | 1,057,570 – 1,615,108 | 180,169 (26.0%) | 108,756 – 251,582 |
| Other | 638,049 (16.5%) | 464,543 – 811,555 | 48,269 (16.2%) | 17,529 – 79,009 | 1,112,340 (13.8%) | 869,805 – 1,354,875 | 112,268 (16.2%) | 56,740 – 167,796 |
| No insurance | 414,711 (10.7%) | 285,330 – 544,092 | 27,800 (9.4%) | 7,135 – 48,465 | 822,263 (10.2%) | 638,960 – 1,005,566 | 91,949 (13.3%) | 46,759 – 137,139 |
| Health Insurance Subcategories (individuals ≥ 65 years of age) | | | | | | | | |
| Private | 1,173,125 (39.9%) | 954,580 – 1,391,670 | 54,076 (22.5%) | 22,105 – 86,047 | 1,682,662 (41.3%) | 1,404,058 – 1,961,266 | 92,281 (21.9%) | 50,599 – 133,963 |
| Dual eligible | 222,034 (7.5%) | 106,298 – 337,770 | 38,353 (16.0%) | 7,496 – 69,210 | 301,094 (7.4%) | 170,725 – 431,463 | 58,721 (14.0%) | 22,531 – 94,911 |
| Medicare only | 1,213,718 (41.3%) | 1,002,657 – 1,424,779 | 120,309 (50.1%) | 61,972 – 178,646 | 1,642,481 (40.3%) | 1,409,694 – 1,875,268 | 216,827 (51.6%) | 136,098 – 297,556 |
| Other | 327,231 (11.1%) | 218,129 – 436,333 | 27,491 (11.4%) | 5,234 – 49,748 | 442,362 (10.9%) | 314,121 – 570,603 | 52,696 (12.5%) | 15,412 – 89,980 |
| Uninsured | 5,420 (0.2%) | -4,875 – 15,715 | NA | NA | 7,365 (0.2%) | -3,385 – 18,115 | NA | NA |
| Preventive Care | | | | | | | | |
| Does not get preventive care | 472,357 (50.6%) | 314,532 – 630,183 | 21,559 (49.0%) | -2,802 – 45,920 | 841,459 (49.4%) | 646,970 – 1,035,948 | 70,472 (41.1%) | 19,258 – 121,686 |
| Clinic or health center | 122,131 (13.1%) | 49,273 – 194,989 | 6,377 (14.5%) | -6,122 – 18,876 | 184,485 (10.8%) | 93,694 – 275,276 | 21,964 (12.8%) | -3,309 – 47,237 |
| Doctor’s office or HMO | 205,638 (22.0%) | 104,529 – 306,747 | 3,506 (8.0%) | -1,790 – 8,802 | 444,372 (26.1%) | 295,210 – 593,534 | 39,365 (23.0%) | 3,398 – 75,332 |
| Hospital emergency room | NA | NA | 6,927 (15.8%) | -3,188 – 17,042 | 28,798 (1.7%) | -7,983 – 65,579 | 10,334 (6.0%) | -1,786 – 22,454 |
| Hospital outpatient | 6,778 (0.7%) | -5,058 – 18,614 | NA | NA | 6,778 (0.7%) | -5,058 – 18,614 | 12,087 (7.0%) | -11,603 – 35,777 |
| Some other place | 19,859 (2.1%) | -7,806 – 47,524 | NA | NA | 51,455 (3.0%) | 7,205 – 95,705 | 8,474 (4.9%) | -8,135 – 25,083 |
| Does not go to one place most often | 106,263 (11.4%) | 37,653 – 174,873 | 5,609 (12.8%) | -2,677 – 13,895 | 144,600 (8.5%) | 67,701 – 221,499 | 8,760 (5.1%) | -1,575 – 19,095 |
| Reasons for Delaying Medical Care in the Past 12 Months | | | | | | | | |
| Could not get through on phone (Y) | 190,984 (2.8%) | 98,774 – 283,194 | 27,833 (5.2%) | -4,997 – 60,663 | 356,092 (2.9%) | 223,169 – 489,015 | 62,911 (5.7%) | 4,748 – 121,074 |
| Could not get through on phone (N) | 6,627,559 (97.2%) | 6,094,115 – 7161,003 | 508,128 (94.8%) | 408,536 – 607,720 | 11780435 (97.1%) | 11,036,416 – 12,524,454 | 1,039,949 (94.3%) | 893,679 – 1,186,219 |
| Could not get appointment soon enough (Y) | 455,862 (6.7%) | 322,230 – 589,495 | 31,326 (5.8%) | 6 – 62,646 | 801,767 (6.6%) | 613,256 – 990,278 | 115,534 (10.5%) | 41,806 – 189,262 |
| Could not get appointment soon enough (N) | 6,362,681 (93.3%) | 5,856,748 – 6,868,614 | 504,635 (94.2%) | 401,860 – 607,410 | 113,31,110 (93.4%) | 10,609,070 – 12,053,151 | 987,326 (89.5%) | 836,717 – 1,137,935 |
| Wait too long in doctor’s office (Y) | 309,916 (4.5%) | 195,181 – 424,651 | 42,847 (8.0%) | 7,907 – 77,787 | 591,399 (4.9%) | 434,476 – 748,322 | 114,223 (10.4%) | 42,928 – 185,518 |
| Wait too long in doctor’s office (N) | 6,508,627 (95.5%) | 5,982,592 – 7,034,663 | 493,114 (92.0%) | 393,386 – 592,842 | 11,545,128 (95.1%) | 10,816,649 – 12,273,607 | 988,637 (89.6%) | 846,839 – 1,130,435 |
| Not open when you could go (Y) | 98,208 (1.4%) | 39,444 – 156,972 | 8,482 (1.6%) | -1,264 – 18,228 | 350,547 (2.9%) | 216,267 – 484,827 | 37,443 (3.4%) | -1,624 – 76,510 |
| Not open when you could go (N) | 6,720,335 (98.6%) | 6,190,923 – 7,249,747 | 527,479 (98.4%) | 419,311 – 635,647 | 11,785,266 (97.1) | 11,057,902 – 12,512,630 | 1,065,417 (96.6%) | 909,751 – 1,221,083 |
| No transportation (Y) | 227,335 (3.3%) | 101,491 – 353,179 | 50,128 (9.4%) | 5,550 – 94,706 | 439,774 (3.6%) | 277,101 – 602,447 | 122,291 (11.1%) | 47,272 – 197,310 |
| No transportation (N) | 6,591,208 (96.7%) | 6,061,815 – 7,120,601 | 485,833 (90.6%) | 388,777 – 582,889 | 11,696,039 (96.4%) | 10,962,668 – 12,429,411 | 980,569 (88.9%) | 840,111 – 1,121,027 |
